# Autonomous identification of freezing of gait in Parkinson's disease from lower-body segmental accelerometry

**DOI:** 10.1186/1743-0003-10-19

**Published:** 2013-02-13

**Authors:** Steven T Moore, Don A Yungher, Tiffany R Morris, Valentina Dilda, Hamish G MacDougall, James M Shine, Sharon L Naismith, Simon JG Lewis

**Affiliations:** 1 Department of Neurology, Mount Sinai School of Medicine, Human Aerospace Laboratory, 10029, New York, NY, USA; 2 Department of Neurology, Robert and John M. Bendheim Parkinson and Movement Disorders Center, Mount Sinai School of Medicine, 10029, New York, NY, USA; 3 School of Psychology, University of Sydney, Sydney, Australia; 4 Parkinson's Disease Research Clinic, Brain and Mind Research Institute, University of Sydney, Sydney, Australia

**Keywords:** FOG, Timed up-and-go task, Accelerometer

## Abstract

**Background:**

We have previously published a technique for objective assessment of freezing of gait (FOG) in Parkinson's disease (PD) from a single shank-mounted accelerometer. Here we extend this approach to evaluate the optimal configuration of sensor placement and signal processing parameters using seven sensors attached to the lumbar back, thighs, shanks and feet.

**Methods:**

Multi-segmental acceleration data was obtained from 25 PD patients performing 134 timed up and go tasks, and clinical assessment of FOG was performed by two experienced raters from video. Four metrics were used to compare objective and clinical measures; the intraclass correlation coefficient (ICC) for number of FOG episodes and the percent time frozen per trial; and the sensitivity and specificity of FOG detection.

**Results:**

The seven-sensor configuration was the most robust, scoring highly on all measures of performance (ICC number of FOG 0.75; ICC percent time frozen 0.80; sensitivity 84.3%; specificity 78.4%). A simpler single-shank sensor approach provided similar ICC values and exhibited a high sensitivity to FOG events, but specificity was lower at 66.7%. Recordings from the lumbar sensor offered only moderate agreement with the clinical raters in terms of absolute number and duration of FOG events (likely due to musculoskeletal attenuation of lower-limb 'trembling' during FOG), but demonstrated a high sensitivity (86.2%) and specificity (82.4%) when considered as a binary test for the presence/absence of FOG within a single trial.

**Conclusions:**

The seven-sensor approach was the most accurate method for quantifying FOG, and is best suited to demanding research applications. A single shank sensor provided measures comparable to the seven-sensor approach but is relatively straightforward in execution, facilitating clinical use. A single lumbar sensor may provide a simple means of objective FOG detection given the ubiquitous nature of accelerometers in mobile telephones and other belt-worn devices.

## Introduction

Freezing of gait (FOG), a paroxysmal block of movement when initiating gait, turning, or negotiating an obstacle, is often described by the Parkinson's disease (PD) patient as the sensation that their feet are 'stuck to the ground'. FOG is generally regarded as a late feature of PD associated with disease duration and severity [1,2], although some early-stage PD patients (patients not yet administered levodopa [2,3] or with mild symptoms [4]) also experience freezing. FOG is associated with an increased prevalence of falls, loss of independence, and nursing home placement [1].

Clinical management of FOG is limited in large part by the difficult nature of assessing its severity, and subjective measures have dominated the field. The Unified Parkinson’s Disease Rating Scale part 14 *'Freezing When Walking'* (UPDRS 14) [5] rates patients on an ordinal scale from 0 (none) to 4 (frequent falls from freezing) based on clinical history. This was the primary outcome measure (UPDRS 14 ≥1) used in a large study of selegiline as a prophylactic treatment for FOG in early PD [6]. A Freezing of Gait Questionnaire (FOG-Q) [7] and the new FOG-Q (NFOG-Q) [8] have recently been proposed as more sensitive tools to identify FOG behavior and assess the efficacy of interventions [9]. However, neither the FOG-Q nor NFOG-Q score correlated with the severity (frequency or duration) of freezing episodes during actual walking in our recent study of PD patients with self-reported FOG [10]. Clinical evaluation of video recordings of ambulating patients to identify the number of FOG events utilizing one [11-15], two [10,15-19], or three observers [20-23] has emerged as a *de facto* 'gold standard' in the past decade. Our recent study utilizing 10 experienced raters across four leading PD centers assessing videos from 'freezers' found only moderate inter-rater agreement for number of FOG events (intraclass correlation coefficient, ICC=0.6), and intra-rater reliability was remarkably low (mean ICC=0.4) [24].

Current treatment options for FOG are largely ineffective [1]. There is an increased prevalence of freezing in advanced disease and in the clinical 'off' state, highlighting the key role of striatal dopamine depletion in its pathogenesis [6]. Increasing levodopa dosage can reduce the frequency of 'off' (unmedicated) state freezing [1,22], likely by increasing the threshold for FOG to occur without altering the underlying pathophysiology [22]. However, FOG commonly shows only partial response to levodopa [22], and the benefits of increasing levodopa dosage in reducing FOG must be balanced with the increased likelihood of levodopa-induced dyskinesias, also associated with a greater fall risk [25]. Patients may undergo deep brain stimulation surgery to relieve symptoms of 'off' state FOG, although these surgical interventions are currently viewed as a treatment option only in the latter stages of PD [26]. Clinical management of dopaminergic therapy to minimize FOG, as well as the evaluation of new targeted interventions, would benefit from the development of objective, standardized FOG measures capable of monitoring this debilitating symptom in a community setting.

We have previously described a technique for identification of freezing episodes based on the frequency characteristics of vertical leg acceleration [14]. The algorithm is predicated on the observation that FOG entails an increase in high frequency (3-8 Hz) leg movement ('trembling') in the relative absence of lower frequency (0-3 Hz) locomotor activity, and the ratio of power in this 'freeze' and' locomotor' band has proven a strong marker of FOG [14]. The original study was based on a small PD patient cohort (N=11) and utilized a single accelerometer on the left shank. In the current study we extend this technique to evaluate the effects of sensor location (back, thighs, shanks, feet) and signal processing parameters (sampling window width and the 'freeze' threshold) in a larger cohort of 'freezers' (N=25) to determine the optimal configurations for autonomous FOG identification from lower body accelerometry.

## Methods

### Recruitment

Twenty five patients (17 male, 8 female) who were attending the Parkinson’s Disease Research Clinic at the Brain and Mind Research Institute were identified for this study by self-reported freezing behavior. All patients satisfied UKPDS Brain Bank criteria [27], had a Mini-Mental State Examination (MMSE) [28] score of ≥ 24 and were deemed unlikely to have dementia or major depression according to DSM-IV criteria by consensus rating of a neurologist (SJGL) and a neuropsychologist (SLN). The study was approved by The University of Sydney Human Research and Ethics Committee and written informed consent was obtained.

### Clinical evaluation and questionnaires

Patients were assessed in the clinically-defined ‘off’ state following overnight withdrawal of dopaminergic therapy. Six patients also had Deep Brain Stimulation (five participants with electrodes implanted in the subthalamic nucleus and one subject with electrodes in the pedunculopontine nucleus), which was turned off 60-min prior to assessment. Patient characteristics were as follows; mean age 69.0 [SD 8.4], disease duration 10.5 years [SD 8.2], Hoehn and Yahr stage [29] 2.7 [SD 0.5], UPDRS – Section III 41.3 [SD 12.3], Parkinson's Disease Questionnaire (PDQ) 39 [30] 67.4 [SD 23.5], and NFOG-Q 18.2 [SD 5.9]. None of the patients described any increase in freezing behavior following the administration of their usual dopaminergic therapy.

### Locomotor task

**Figure 1:**
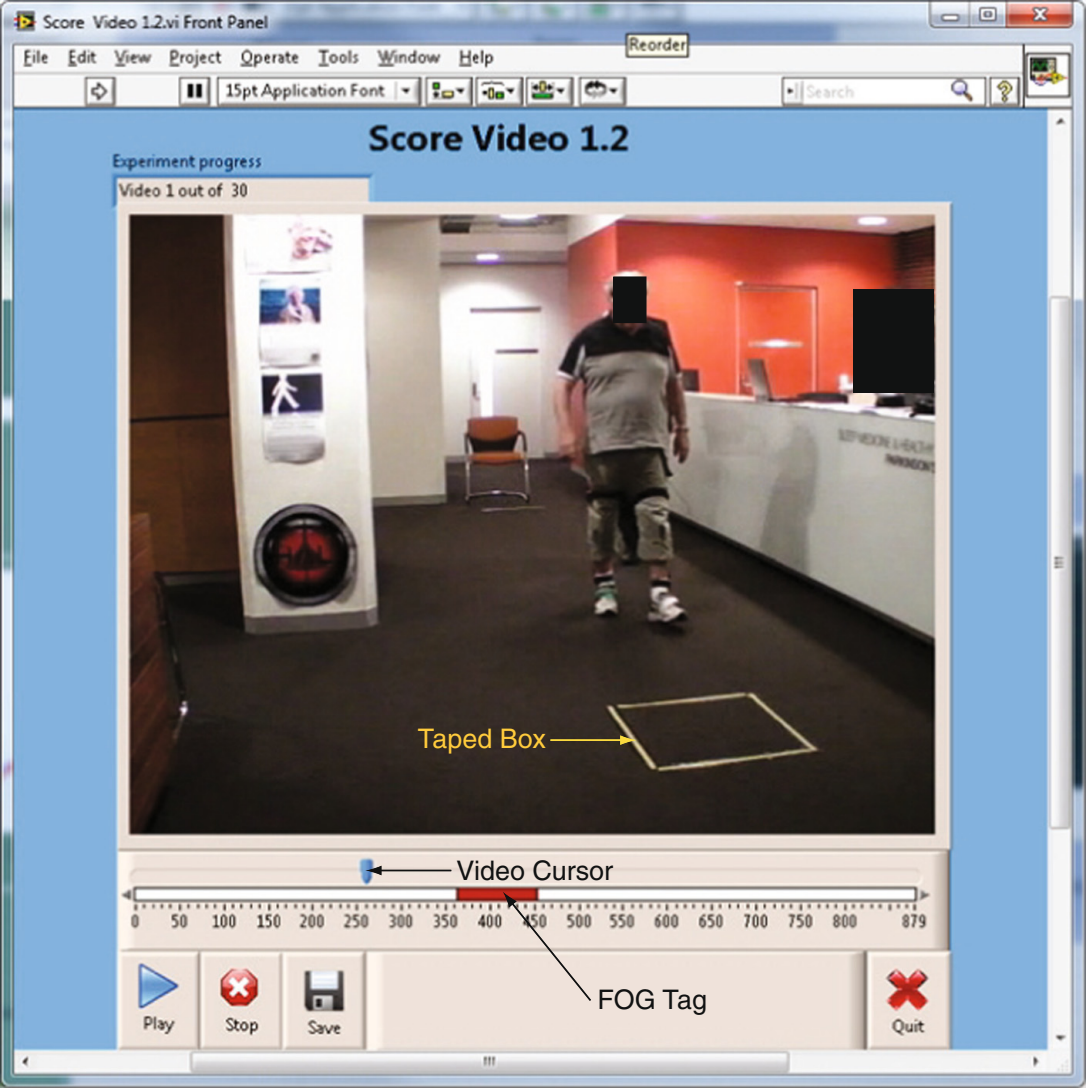
**Screen image of the FOG tagging software developed by the investigators.** The video image shows a PD patient performing the timed up-and-go (TUG) task, in which subjects start from a seated position and walk 5 m to a target box marked on the floor with tape, execute a turn, and return to the seated position. Clinicians reviewed the video and indicated a FOG event by holding down the 'T' key, resulting in a FOG tag (red rectangle) which could be resized by dragging the ends horizontally in time, which moved the video by a corresponding amount. The video cursor could also be dragged forwards and backwards in time to facilitate repeat viewing of FOG events.

The 25 patients performed a total of 134 timed up-and-go (TUG) tasks (mean 5.4 [SD 1.7] per subject) to provoke FOG on a standardized 5-m course [10,24]. Participants, starting from a seated position, walked 5 m to a 0.6 × 0.6 m box marked on the floor with yellow tape, in which a turn to the left or right was performed before returning to the chair (Figure 1). Walking trials were recorded on a digital video camera from a consistent vantage point for later analysis [10,24], and each video showed a complete TUG trial starting and ending in the seated position.

### Data acquisition

**Figure 2:**
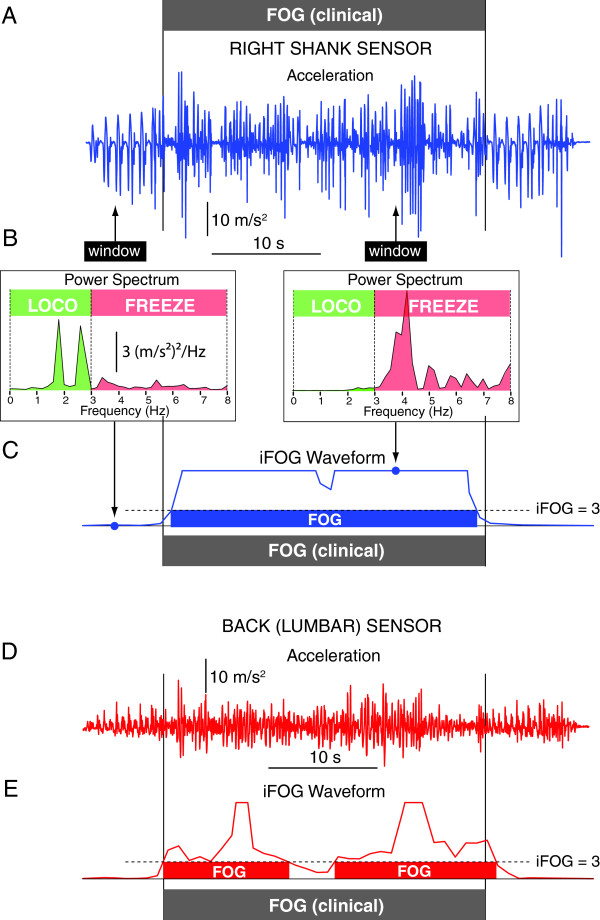
**A Vertical acceleration data from the right shank during a TUG task of 43-s duration.** The grey rectangle indicates FOG as determined by the clinical raters. **B** At each point in time acceleration data was sampled from a window (black rectangle - set to a width of 5 s in this instance) and a power spectrum calculated (inset). The ratio of power (area under the curve) in the freeze (3-8 Hz) and locomotor (0-3 Hz) bands from these spectra was determined to form an index of FOG (iFOG). **C** The iFOG waveform (shown here truncated at 10) was used to identify freezing using a threshold (set to 3 in this example); any periods in which the iFOG waveform was above threshold were tagged as a FOG event. **D** Vertical acceleration from the lumbar sensor was attenuated relative to the shank but still exhibited similar high frequency characteristics, enabling FOG detection **E**.

Subjects were instrumented with seven inertial measurement units (IMUs - Xsens MTx, Enschede, Netherlands) secured to the lumbar region of the back (approximately L2), the lateral aspect of each thigh and shank, and the superior aspect of each mid-foot, with elasticized straps such that the sensors were affixed to the segment as a rigid body. The IMUs were small (38 × 53 × 21 mm; 30 g) and did not interfere with natural movement. During testing each IMU acquired triaxial linear acceleration (only longitudinal acceleration of each body segment was considered in this study; Figure 2A,D), transmitted wirelessly to a computer at a sample rate of 50 Hz). Synchronization of the video and accelerometer recordings was performed prior to data collection by alignment of the video camera and data-acquisition computer clocks.

### Objective assessment of FOG

We have previously described a technique for objective identification of freezing episodes based on the frequency characteristics of vertical shank acceleration from a single sensor [14], which is summarized here. Peaks at the stride and step frequencies (approximately 1 and 2 Hz, respectively [31]) characterize the power spectrum of vertical leg acceleration during an epoch of walking, with the vast majority of power occurring at frequencies below 3 Hz [14]. Thus 0-3 Hz was defined as the ‘locomotor’ band. In contrast, FOG is characterized by an absence of forward gait and the development of high frequency trembling of the lower limbs, which were found to occur primarily within 3 to 8 Hz [14]; this region was designated the ‘freeze’ band. These characteristic frequency signatures were used to discriminate FOG (during which power is significantly decreased or absent in the locomotor band relative to a large increase in power in the ‘freeze’ band) and normal gait (in which the vast majority of power is in the locomotor band). Specifically, for a particular period of leg acceleration a threshold could be set for the ratio of freeze to locomotor band power above which a particular epoch was identified as a FOG event. This freeze threshold ranged from 2.3 to 4.4 in individual patients [14] (i.e., FOG was associated with freeze band power approximately 2 to 4 times the power in the locomotor band).

Another parameter of interest is the duration of the sampling epoch. In our original study [14] 50% of FOG episodes were found to be less than 10 s in duration, and 15% were under 4 s. Applying the FOG identification algorithm with window sizes >10 s tended to average out short FOG events, effectively acting as a low pass filter [14]. Thus, for this study 10 s was considered the upper limit of sampling window size. A smaller window may be more sensitive to shorter FOG events, whereas a wider window of 10 s may improve specificity. The aim of the current study was to systematically explore the effects of freeze threshold and window size, as well as the use of multi-segmental sensor placement, on the sensitivity and specificity of FOG detection.

**Figure 3:**
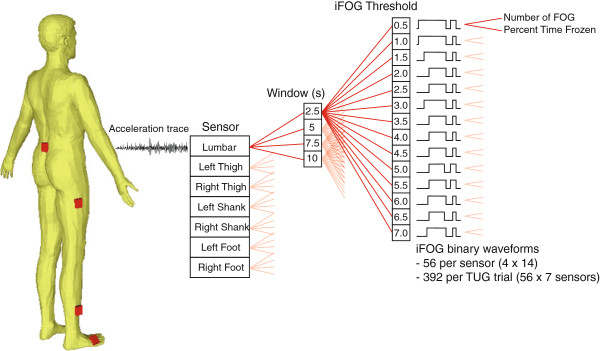
**Visual representation of the dataset generated by a single TUG trial.** Each of the seven sensors generated an acceleration trace; each trace was processed with sampling windows of 2.5, 5, 7.5 and 10 s to generate four iFOG waveforms (the ratio of freeze to locomotor band power); fourteen binary traces from each of the four iFOG waveforms were then formed using an iFOG threshold of 0.5 to 7 in steps of 0.5, resulting in a total of 56 binary FOG traces per sensor, and 392 per TUG trial. Each binary waveform provided a measure of number of FOG events and the percent time frozen.

For each acceleration trace from a TUG task a sampling window (width of 2.5, 5, 7.5 or 10 s), centered on the current data point, was used to obtain a 'snapshot' of acceleration every 200 ms (Figure 2B). A spectral analysis (*auto power spectrum*, Labview, National Instruments, Austin, TX), was performed on the data subset within this window (Figure 2B) and the index of freezing of gait (iFOG) was defined as the ratio between (i) the square of the area under the acceleration power spectrum in a freeze band (3-8 Hz) and (ii) the square of the area under the acceleration power spectrum in a locomotor band (0-3 Hz) (Figure 2B). A discrete freeze event was defined as a contiguous period of time in which iFOG was greater than a defined threshold (Figure 2C,E), which was varied in this study from 0.5 to 7 in steps of 0.5 (i.e., the ratio of freeze to locomotor band power ranged from 1:2 to 7:1). For a particular sampling window width and iFOG threshold the algorithm produced a binary waveform with a baseline of zero and a value of one representing suprathreshold iFOG values (i.e., freezing of gait; Figure 2C,E). This first stage of analysis yielded 56 binary waveforms (for each of the four sampling windows there were 14 waveforms corresponding to the 14 threshold values) per sensor for each TUG trial, from which the *number of FOG events* (iFOG > threshold) and *percent time frozen* (the cumulative duration of all FOG episodes divided by the total duration of the walking task) were calculated (Figure 3). Processing of all 134 TUG trials resulted in an array for number of FOG (134 elements) for each window size, threshold value and sensor (4 × 14 × 7, total 392 arrays); similarly, 392 134-element arrays were formed for percent time frozen (Figure 3).

The results of autonomous FOG identification from combinations of sensors were compared in post-hoc analysis. A majority vote algorithm was used to identify FOG using all seven IMUs (back, thighs, shanks and feet); at each point in time the combined binary FOG waveform was set to '1' if 4 or more sensors indicated a freeze event (and '0' if less than 4 sensors registered FOG). FOG identification from each limb segment (thighs, shanks, feet) utilized a logical ‘OR’ operation; if the iFOG ratio exceeded the freeze threshold in either the left or right sensor (or occurred in both sensors) a freeze event was flagged. The ability of the single lumbar sensor to discriminate FOG was also evaluated. In addition, FOG identification from individual shank IMUs (left or right) was assessed to allow comparison with our previous study [14], which utilized a single sensor on the left shank. (Freeze identification from individual thigh and feet sensors were also assessed, but are not discussed in the manuscript to reduce complexity; for completeness, results from individual thigh and foot sensors are included as Additional file 1).

### Clinical assessment of FOG

The frequency and relative duration of freezing episodes were determined for each video utilizing a FOG tagging program described previously [10,24]. Two clinicians experienced in FOG (JMS and SJGL) used their best clinical judgment to identify FOG episodes from video, tagging the onset of a freeze by pressing the ‘T’ key and holding down the key for the duration of each event (Figure 1). Video editing tools enabled the ends of a horizontal bar representing the duration of each tagged freeze to be dragged backwards or forwards in time to facilitate accurate logging of FOG onset and offset. Analogous to the accelerometer processing, clinical ratings were saved as a binary signal at the video frame rate (30 Hz), with a zero baseline and 1 indicating freezing of gait. The results from the two raters were combined using a logical OR operation, resulting in a single binary FOG waveform for each TUG task. The number of FOG events and the percent time frozen were calculated from this signal (as described above), resulting in two arrays (number of FOG; percent time frozen) of 134 elements (corresponding to the 134 TUG trials). This rater pairing achieved high agreement (intraclass correlation coefficient [ICC] for number of FOG=0.82; percent time frozen ICC=0.99) in a previous study using this scoring technique [24].

### Statistical analysis

The reliability of the quantification of the number of FOG episodes and percent time frozen was calculated between the clinical observers and the objective accelerometer-derived (iFOG) measures using the ICC [32]; the 134-element arrays for frequency, and relative duration of FOG, obtained from clinical assessment were compared to each of the 392 arrays representing the full range of window size (n=4), iFOG thresholds (n=14) and sensor locations (n=7). We used the following classification of ICC power: <0.2 negligible, 0.2≤0.4 weak, 0.4≤0.7 moderate, 0.7≤0.9 strong, and >0.9 very strong [24]. Sensitivity-specificity analysis assessed the performance of each sensor group as a binary classification test, assuming that the subjective clinical ratings were a true 'gold standard'. For each of the 134-element arrays, sensitivity was defined as the ratio of the number of trials having at least one FOG event as determined from objective acceleration data over the number of trials having at least one FOG event as identified by the clinical raters. Similarly, specificity was defined as the ratio of the number of trials having no FOG activity as determined from objective acceleration data over the number of trials having no FOG activity as identified by the clinical raters.

## Results

Twenty out of the twenty-five participants exhibited clinically identifiable FOG during the study. A total of 298 FOG events (range 0-50 per subject; mean 11.9 [S.D. 13.4]) were identified from the video recordings by the clinical raters; percent time frozen averaged 24.1% [SD 24.8] (range 0-72.4%). The frequency and relative duration of FOG determined by the sensors was dependent on three parameters; 1) the sensor location(s), 2) the size of the sampling window, and 3) the iFOG threshold. For example, with a sampling window of 5s and an iFOG threshold of 3 (as shown in Figure 2) the results from all seven sensors (majority vote) were a total of 354 FOG events (range 0-59 per subject; mean 13.9 [S.D. 13.3]), and percent time frozen averaged 22.7% [SD 18.4] (range 0 - 66.3%).

### Number of FOG

**Figure 4:**
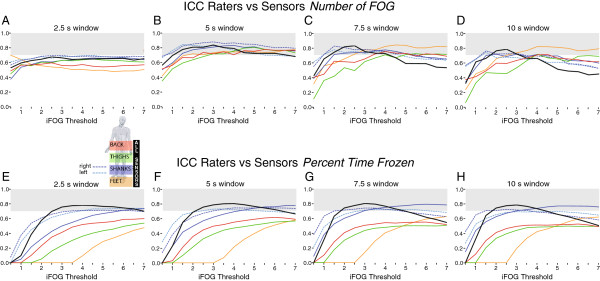
Agreement between clinical assessment and accelerometry (as measured by the intraclass correlation coefficient, ICC) for number of FOG events (A-D) and percent time frozen (E-H), as a function of sensor location, sampling window width, and threshold.

A window size of 5 s provided the strongest agreement between raters and sensors for number of FOG, with all sensors achieving an ICC>0.7 at iFOG thresholds of 3 or greater (Figure 4B). A 2.5 s window size produced only moderate agreement (ICC<0.7), with the best performing locations being all seven IMUs (majority vote) and the individual left and right shank sensors, with ICCs greater than 0.6 over almost the entire range of thresholds (Figure 4A). All seven sensors, and the individual shank sensors, exhibited strong agreement (ICC>0.7) with the raters at window sizes of 7.5 and 10 s within a narrow threshold range of 1.5-3 (Figure 4C,D).

### Percent time frozen

The agreement between clinicians and sensors for percent time frozen was essentially independent of the width of the sampling window (Figure 4E-H). The best performing locations were all seven IMUs (majority vote) and the individual left and right shank sensors, with ICCs>0.7 within a threshold range of 2-4.

### Sensitivity and specificity

**Figure 5:**
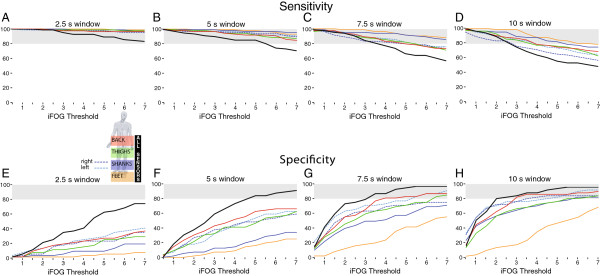
Sensitivity (A-D) and specificity (E-H) of accelerometer-based detection of FOG, as a function of sensor location, sampling window width, and threshold.

The sensitivity of the sensors to FOG events was inversely related to sampling window size. Within a small (2.5 or 5 s) window (Figure 5A,B) all sensor combinations exhibited sensitivity above 70% over the entire range of thresholds. Sensitivity tended to drop off above a threshold of 3 as window size increased to 7.5 and 10 s (Figure 5C,D). In contrast, increased specificity was strongly related to larger window size (Figure 5E-H). Not surprisingly, the most specific sensor combination was all seven IMUs (majority vote), achieving specificity above 80% for windows of 7.5 and 10 s and thresholds ≥ 3. The single lumbar sensor exhibited a robust sensitivity and specificity (70-80%) within a range of thresholds of 3.5-5 at the larger window sizes (7.5 and 10 s), closely followed by the individual shank sensors (Figure 5G,H). The feet sensors were very sensitive to FOG events but had the lowest specificity (Figure 5E-H).

Based on these results we recommend three optimal configurations for autonomous FOG monitoring (Table 1).

**Table 1:** Recommended configurations for autonomous FOG identification from accelerometry with corresponding performance metrics

**System**	**# Sensors**	**Threshold**	**Window**	**ICC number of FOG**	**ICC percent time frozen**	**Sensitivity**	**Specificity**
'reference'	7	3	7.5 s	0.75	0.80	84.3	78.4
shank (L or R)	1	3	7.5 s	0.75	0.73	86.2	66.7
back (lumbar)	1	3	10 s	0.63	0.49	86.8	82.4

## Discussion

The results from this study demonstrate the feasibility of objective monitoring of FOG from lower body accelerometry, and provide a practical basis for implementation based on the particular goal of the application. Three parameters must be considered; the location and number of sensors, the width of the sampling window for determining the ratio of freeze (3-8 Hz) to locomotor (0-3 Hz) band power at each point in time, and the threshold above which this ratio indicates a FOG event. These choices represent tradeoffs between sensitivity and specificity; the more distal the sensor, the narrower the window, and the lower the threshold, the higher the sensitivity of FOG detection at the expense of specificity (more false positives).

The use of seven sensors (back, thighs, shanks and feet) and a majority vote (at least 4 sensors registering FOG) provided the most consistently robust measure of freezing across the range of window and threshold values, and should be considered the 'reference' system against which other combinations of sensors are judged. A close second was the use of individual left or right shank accelerometers, which suggests that the 'trembling' associated with FOG is a bilateral ‘bottom-up’ phenomenon (higher power distally – see also Figure 2). A threshold of 3 was found to produce strong agreement with the clinical raters (ICC>0.7) for individual shank sensors at window sizes 5 s and above. This is consistent with our original study [14], in which the optimal individual threshold for a sensor on the left shank ranged from 2.3 to 4.4 at a window size of 6 s. At low FOG thresholds (<3) the ICC for percent time frozen was higher for individual shank sensors than the combined shanks (Figure 4). This was likely due to increased false positives in individual shank sensors when the threshold was set too low; logically combining these FOG measures lowered the agreement with the clinical observers. For iFOG thresholds ≥ 3 the combined shank ICC was consistent with single shank sensors. The single lumbar sensor provided sensitivity and specificity over 80% with a threshold of 3 and a window size of 10 s; although the high frequency lower-limb 'trembling' of FOG was attenuated by the musculoskeletal system relative to the more distal sensors (Figure 2A,D), sufficient power was present to reliably identify the presence of FOG.

Objective identification of the number of FOG events tended towards overestimation relative to the clinical observers; for example, using seven sensors with a 5 s second window and an iFOG threshold of 3 identified 354 discrete FOG events from the 134 TUG trials, 19% higher than the 298 episodes observed by the clinicians. This may be due to the ability of accelerometry to detect subclinical variations in trembling intensity not visible to the naked eye, resulting in a higher FOG count. Moreover, our recent work [10,24] has demonstrated that percent time frozen is a more robust clinical metric of FOG severity than simply counting the number of freeze events, as it minimizes individual differences in scoring multiple sequential, or one longer, FOG event(s). Agreement (ICC) between 10 movement disorder specialists scoring a set of 14 videos of patients performing the TUG task was 16% higher for percent time frozen relative to number of FOG [24] (our two raters demonstrated a similar relative agreement in the same study, with an ICC of 0.82 for number of FOG and 0.99 for percent time frozen). In the current study, mean percent time frozen (all seven sensors; 5s window, iFOG threshold 3) per subject from accelerometry (22.7%) and clinical observation (24.1%) was in close agreement.

The results of this study demonstrate that objective FOG identification based on the frequency characteristics of lower body motion can achieve strong agreement (ICC > 0.7) with clinical assessment by movement disorder specialists. Advances in motion capture hardware may reduce the size and complexity of multi-segmental accelerometry, facilitating use in the community. Increasing the agreement between objective and subjective measures may require more than technical enhancement, given the current lack of standardized criteria for determining FOG onset and offset [24]. The accelerometer-based approach provides the advantage of an adjustable, objective criteria for FOG onset and offset based on the frequency characteristics of lower-limb tremor; this may prove of benefit when training movement disorder specialists in clinical assessment of freezing, providing feedback on the nature of trembling associated with FOG.

## Conclusions

Evaluation of FOG in clinical practice is notoriously difficult due to its paroxysmal nature, and current subjective approaches lack utility. There is a clear need for the development of objective standardized measures for assessment of future pharmacological and non-pharmacological interventions, and the results presented herein provide a practical basis for the implementation of accelerometry-based FOG monitoring. The seven-sensor approach was both the most complex and most robust system, scoring highly on all measures of performance, and is better suited to demanding research applications. A single shank sensor provided FOG measures comparable to the seven-sensor approach but is considerably simpler in execution, facilitating clinical use. A single lumbar sensor exhibited only moderate agreement with the clinical raters in terms of absolute number and duration of FOG events but had high sensitivity and specificity as a binary test of FOG, and lends itself readily to ambulatory monitoring given the ubiquitous nature of accelerometers in mobile telephones and other belt-worn devices.

## Competing interests

The authors declare that they have no competing interests.

## Authors’ contributions

The study was designed by STM and SJGL. STM designed and implemented algorithms and software for processing of lower-body acceleration data to identify FOG events, analyzed data, and wrote the manuscript; DAY implemented software for automated data analysis and analyzed data; STM, TRM, VD, JMS, SLN and SJGL participated in data collection. All authors reviewed and approved the final manuscript.

## Supplementary Material

Additional file 1 Agreement between clinical assessment and accelerometry (as measured by the intraclass correlation coefficient, ICC) for number of FOG events and percent time frozen for individual (right and left) thigh and feet sensors. Click here for file
